# When Stress Is Not the Only Culprit: A Case of Secondary Adrenal Insufficiency in a Young Patient With Graves’ Disease

**DOI:** 10.7759/cureus.41528

**Published:** 2023-07-07

**Authors:** Saleha Ozair, Hiram Maldonado-Rivera, Kimberly Medina-Morales, Echols Marti, Eugenio Angueira-Serrano, George Michel

**Affiliations:** 1 Internal Medicine, Larkin Community Hospital, South Miami, USA; 2 Endocrinology, Larkin Community Hospital, South Miami, USA; 3 Research, Larkin Community Hospital, South Miami, USA; 4 Endocrinology, Larkin Community Hospital Palm Springs Campus, Hialeah, USA

**Keywords:** graves' disease, t3-thyrotoxicosis, addisonian crisis, hypotension, secondary adrenal insufficiency

## Abstract

This case report underscores the significance of maintaining a broad list of differential diagnoses, including adrenal insufficiency, when evaluating patients who present with recurring episodes of hypotension and generalized fatigue. It further underscores that T3 thyrotoxicosis can manifest as the initial and sole presenting feature of Graves' disease. Finally, it emphasizes the critical importance of employing a multidisciplinary approach to discharge high-risk patients from the hospital to minimize the risk of acute decompensation.

## Introduction

Adrenal insufficiency (AI) can arise from primary adrenal disease or impaired hormonal signaling to the adrenal gland, referred to as secondary adrenal insufficiency (SAI) [1]. The symptoms of SAI are nonspecific, with an insidious onset that poses a diagnostic challenge. About one-half of all patients have symptoms for over one year, and the majority see three or more physicians prior to diagnosis [2,3]. If left untreated, AI can lead to chronic fatigue, weight loss, and increased susceptibility to infections, with a life-threatening adrenal crisis being a possible outcome [4]. Timely recognition and management of AI is therefore critical to improving the patient's quality of life, preventing acute crises, and reversing chronic ill health. There is still considerable debate regarding the most effective therapy for SAI, with limited data available on the comparative efficacy of dual-release hydrocortisone versus modified-release glucocorticoids [5]. Retrospective studies have demonstrated that up to 44% of patients with AI have experienced an adrenal crisis at least once during their lifetime, highlighting the importance of patient education in managing this chronic condition [6]. In this case report, we present a 34-year-old female with recurrent hypotensive episodes and generalized fatigue, who was subsequently diagnosed with SAI and required readmission within three days due to non-procurement of the prescribed medications leading to adrenal crisis.

## Case presentation

A 34-year-old female from Macedonia with no significant past medical history presented to the hospital for generalized fatigue and several documented episodes of hypotension, measuring 70/40 in the mornings. The patient reported experiencing worsening of exertional shortness of breath, palpitations, orthopnea, and dyspnea for the past one week, which she attributed to workplace stress. The patient also endorsed myalgia in the distal muscles of her upper and lower extremity, which she attributed to her mechanical fall one year ago. The myalgia worsened with use. The patient stated that she had been requiring three pillows to sleep comfortably, occasionally felt dizzy, and spent most of her day sleeping as she felt weaker than usual. Previously, she used to be able to climb five flights of stairs but now had increased fatigability with just walking around the hotel lobby and was unable to get out of bed the whole day due to lethargy. The patient also stated that three days ago, she had a blood pressure of 60/30 but denied having any syncopal episodes, loss of consciousness, hypoglycemic episodes, nausea, vomiting, or abdominal pain. The patient endorsed having heat intolerance, recurrent hair loss, and loose stools for the past month but denied having any weight loss, change in menstrual periods, or any other symptoms of thyroid dysfunction. Initial workup revealed thyroid-stimulating hormone (TSH) of 0.18 mlU/mL (L), free T4 was 1.4 ng/dL (within normal limits (WNL)), total T3 was 1.3 ng/mL (WNL), and free T3 was 11.10 pmol/L (H). Findings were suggestive of T3 thyrotoxicosis. The anti-thyroid peroxidase (anti-TPO) level was 11 IU/mL (WNL) and thyrotropin receptor antibody (TRAb) was 1.10 IU/L (WNL), which ruled out Hashimoto's disease as a cause of thyrotoxicosis. Table 1 further itemizes each thyroid hormone level during her hospitalization.

**Table 1:** Thyroid test results

Test	Results	Reference range
Thyroid-stimulating hormone (TSH)	0.18 mlU/mL	0.4-4.0 mlU/mL
Free T4	1.4 ng/dL	0.9-1.7 ng/dL
Total T3	1.3 ng/dL	0.92-2.76 ng/dL
Free T3	11.10 pmol/L	3.0-7.0 pmol/L
Anti-thyroid peroxidase (anti-TPO)	11.0 IU/mL	<15.0 IU/mL
Thyrotropin receptor antibody (TRAb)	1.10 IU/mL	1.0-1.8 IU/mL

**Figure 1:**
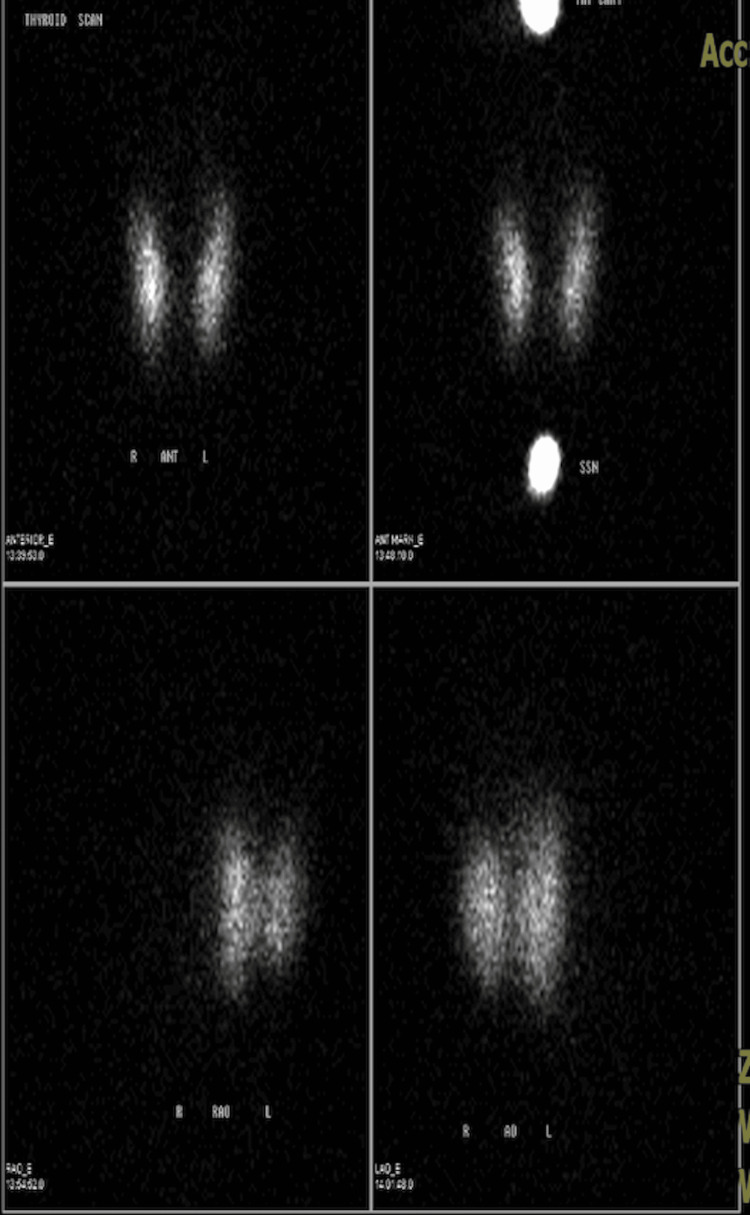
Radioactive iodine uptake (RAIU) scan showing homogeneously increased thyroid uptake of 42%, suggestive of Graves' disease

Radioactive iodine uptake (RAIU) scan showed homogeneously increased thyroid uptake, suggestive of Graves' disease (Figure 1). Thyroid-stimulating immunoglobulin (TSI) was <0.10%; however, a diagnosis of Graves' disease was confirmed due to radiological evidence of an overactive thyroid and T3 thyrotoxicosis, which can be one of the earliest signs of Graves' disease. As such, the patient was started on methimazole 5 mg oral (PO) every other day (QOD) and advised to repeat the thyroid panel in six weeks. The patient had also tested positive for orthostatic hypotension and random cortisol was 4.5 (inappropriately low). Adrenocorticotropic hormone (ACTH) stimulation test resulted in AM cortisol of 2.0 ug/dL, 18.2 ug/dL 30 minutes after the test, and 21.8 ug/dL 60 minutes later. This ruled out primary adrenal insufficiency; however, the patient was worked up for SAI due to the low AM cortisol level. Morning (8 AM) ACTH level was 7.8 pg/mL (L), which confirmed a diagnosis of SAI, and the patient was started on a stress dose of steroids (hydrocortisone 25 mg PO three times a day (TID)), which was tapered down to 20 mg PO once a day in the morning (QAM) and 10 mg PO once a day in the evening (QPM), once the adequate response was achieved. The patient was also started on midodrine 5 mg PO TID in the interim to prevent further episodes of hypotension. Notably, no electrolyte abnormalities were present. Further workup was ordered to determine the etiology of SAI and given that both TSH and ACTH were low, other pituitary hormones were tested to rule out a pituitary defect. Prolactin was 47.5 ng/mL (H), luteinizing hormone (LH) level was 2.5 IU/L (low normal), growth hormone was 0.4 ng/mL (low normal), insulin-like growth factor 1 (IGF-1) was 262 ng/mL (WNL), and follicle-stimulating hormone (FSH) was 3.4 mIU/mL (WNL). Table 2 further itemizes each cortisol and pituitary hormone level during her hospitalization.

**Table 2:** Cortisol and pituitary hormones test results ACTH: adrenocorticotropic hormone; LH: luteinizing hormone; FSH: follicle-stimulating hormone; IGF-1: insulin-like growth factor 1.

Test	Results	Reference range
Random cortisol	4.5 mcg/dL	5.0-25.0 mcg/dL
ACTH stimulation test	AM: 2.0 ug/dL, 30 min after: 18.2 ug/dL, 60 min after: 21.8 ug/dL	5.0-25.0 ug/dL
Morning ACTH (8:00 AM)	7.8 pg/mL	5.0-25.0 pg/mL
Prolactin	47.5 ng/mL	<25.0 ng/mL (females)
LH	2.5 IU/L	5.0-20.0 IU/L
FSH	3.4 mIU/mL	1.4-9.9 mIU/mL (follicular phase), 6.2-17.2 mIU/mL (ovulatory phase), 1.1-9.2 mIU/mL (luteal phase)
Growth Hormone	0.4 ng/mL	0.4-10.0 ng/mL
IGF-1	262.0 ng/mL	182.0-780.0 ng/mL

Brain MRI showed no evidence of macroadenoma, empty-sella syndrome, or lymphocytic hypophysitis; however, it did show a pineal cyst, measuring approximately 10.1 x 9.1 x 7.9 mm, which was deemed to be clinically insignificant and the patient was advised to follow up annually with neurosurgery service. Of note, the patient denied consuming any exogenous steroids or opiates but does report a strong family history of autoimmune conditions. The importance of medication compliance and regular follow-up with endocrinologist and primary care physician post-discharge was reiterated. The patient was also advised to wear a medical ID bracelet and educated regarding the administration of stress doses of steroids in case of an emergency like an Addisonian crisis. Despite multiple attempts to educate the patient’s workplace staff regarding the importance of medication compliance, challenges were encountered in providing the patient with a sufficient supply of all required meds due to logistic and administrative barriers, which led to re-hospitalization for hypotensive crisis within three days from being discharged. The patient was, however, hemodynamically stable once she received her scheduled hydrocortisone dose and 1 L bolus of IV fluids (crystalloids). This underscores the importance of close collaboration between physicians, nurses, pharmacists, case managers, and patient’s workplace staff in planning safe discharges and avoiding potential complications.

## Discussion

SAI is typically characterized by weight loss, fatigue, and myalgia [4]. Some of the common presenting signs of SAI include alabaster skin, euvolemia, normotension, hyponatremia, normokalemia, hypoglycemia, normal calcium, and central hypothyroidism [4]. In this case, the patient did not exhibit any of the typical signs, and the diagnosis was challenging. Pallor/alabaster skin color was difficult to appreciate as the patient was from Macedonia and was genetically light-skinned. However, once the diagnosis was made, further investigation to ascertain the underlying etiology was performed. The most common endogenous cause of SAI is a tumor of the hypothalamic-pituitary region [7], and common exogenous causes include prolonged steroid or opioid use. Less commonly, SAI can be caused by infiltrative lesions, infective processes, vascular alterations, traumatic brain injury, or genetic disorders. Addison's disease is frequently a component of autoimmune polyendocrinopathies, but SAI is rarely associated with autoimmune disorders [8]. In the presence of a strong family history of autoimmune conditions, patients should be evaluated for lymphocytic hypophysitis as a cause of SAI. Kasperlik-Załuska et al. conducted a case series on a group of 102 patients with secondary adrenal failure of unknown origin and noted that the most frequently associated autoimmune disorder was thyroid abnormalities [8]. Other diseases included insulin-dependent diabetes mellitus, pernicious anemia, vitiligo, premature ovarian failure, and autoimmune thrombocytopenia [8]. Thyroid autoimmunity was manifested by overt or subclinical hypothyroidism, thyrotoxicosis, and/or the presence of circulating antithyroid antibodies [8]. Another case series conducted by Michael Karl et al. concluded that symptomatic hypocortisolemia may be present in severe hyperthyroidism, and it resolves with adequate treatment of the hyperthyroidism. A review of existing literature showed that there is considerable evidence that the hyperthyroidism associated with Graves' disease is often times accompanied by T3 thyrotoxicosis [9,10]. In this particular case, the patient displayed symptoms of tachycardia at night, night sweats, and low-grade fever. As a result, a diagnosis of Graves' disease was confirmed based on radiological evidence indicating an overactive thyroid and T3 thyrotoxicosis. Graves' disease, an autoimmune condition, primarily affects the thyroid gland and presents with symptoms such as tremors, heightened sensitivity to heat, increased warmth, unexplained weight loss despite normal eating habits, anxiety, and irritability [11].

## Conclusions

In conclusion, although our patient's symptoms were initially vague and could be attributed to other factors, a high level of clinical suspicion supported by positive orthostatic vitals, recurrent hypotensive episodes, and a new diagnosis of Graves' disease led to the timely and accurate recognition of SAI. The patient received appropriate medical supplementation and will require frequent monitoring of symptoms and laboratory tests to guide necessary adjustments to the current treatment plan. It is absolutely crucial for such patients to remain vigilant about their risk of going into an adrenal crisis.
